# Preliminary results of abdominal simultaneous multi-slice accelerated diffusion-weighted imaging with motion-correction in patients with cystic fibrosis and impaired compliance

**DOI:** 10.1007/s00261-022-03549-7

**Published:** 2022-05-21

**Authors:** Katja Glutig, Paul-Christian Krüger, Theresa Oberreuther, Marcel Dominik Nickel, Ulf Teichgräber, Michael Lorenz, Hans-Joachim Mentzel, Martin Krämer

**Affiliations:** 1 Department of Radiology, Section Pediatric Radiology, Jena University Hospital, Am Klinikum 1, 07747 Jena, Germany; 2 Department of Radiology, Jena University Hospital, Jena, Germany; 3 Cystic Fibrosis Centre, Department of Paediatrics, Jena University Hospital, Jena, Germany; 4 MR Applications Predevelopment, Siemens Healthcare GmbH, Erlangen, Germany

**Keywords:** ADC value, Motion robustness, Signal-to-noise-ratio, Free-breathing, Scan time

## Abstract

**Objectives:**

The aim of this prospective study was to compare scan time, image quality, signal-to-noise Ratio (SNR), and apparent diffusion coefficient (ADC) values of simultaneous multi-slice accelerated diffusion-weighted imaging with motion-correction (DWI SMS Moco) to standard diffusion-weighted imaging (sDWI) in free-breathing abdominal magnetic resonance imaging (MRI) in pediatric and young adult patients with cystic fibrosis (CF).

**Material and methods:**

16 patients (7 male and 9 female, 12–41 years old) with CF were examined prospectively in a single-center from November 2020 to March 2021 on a 1.5 Tesla clinical MR scanner. The characteristics of overall image quality and delimitability of mesenteric lymph nodes were evaluated using a 5-point Likert scale by two experienced pediatric radiologists independently from each other. Quantitative parameters with SNR and ADC values were assessed in 8 different locations and compared using a Wilcoxon signed-rank test.

**Results:**

The acquisition time for DWI SMS Moco was 32% shorter than for sDWI. Regarding quality comparison, overall image quality and delimitability of mesenteric lymph nodes were significant higher in DWI SMS Moco (*p* ≤ 0.05 for both readers). The readers preferred DWI SMS Moco to sDWI in all cases (16/16). Mean SNR values from DWI SMS Moco and sDWI were similar in 7 from 8 locations. The ADC values showed no significant difference between DWI SMS Moco and sDWI in any of the evaluated locations (*p* > 0.05).

**Conclusions:**

The DWI SMS Moco improves overall image quality and delimitability of mesenteric lymph nodes compared to sDWI with similar SNR and ADC values and a distinguished reduction of scan time in free-breathing by one third. We conclude that MRI with DWI SMS Moco could be helpful in monitoring the effect of the high-efficiency modulator (HEM) therapy in cystic fibrosis (CF) patients homozygous or heterozygous for F508del in the abdomen.

## Introduction

Diffusion-weighted imaging (DWI) is routine in central nervous system (CNS) diagnostics [1], whole-body imaging (WBI) [2], in oncological questions [3] and suspected inflammatory processes [4]. In abdominal magnetic resonance imaging (MRI), diffusion-weighted sequences can reliably detect lesions and provide functional information on tumor tissue, like cellular density [5–7]. Frequently, a DWI with evaluation of the corresponding apparent diffusion coefficient (ADC) is included in most abdominal MRI protocols [8]. However, the use of DWI in abdominal imaging can be problematic due to respiratory motion artefacts, and due to greatly prolonged acquisition times in combination with respiratory gating [9], which is intensified in cystic fibrosis (CF) patients who suffer from irregular breathing patterns in the supine position during abdominal MR examinations [10, 11]. Therefore, reduced acquisition time of DWI scans is of major importance for CF patients.

The simultaneous multi-slice (SMS) acquisition technique was developed to reduce acquisition time by simultaneous excitation and acquisition of multiple slices [12–16]. An additional non-rigid motion correction (Moco) in DWI with SMS technique could optimize image quality [17–19].

Recently, major advances in care and improved therapy with highly effective modulator (HEM) options have reduced the morbidity and mortality of pulmonary involvement of cystic fibrosis significantly [20–23]. CF patients are able to live well into their sixth decade of life [24, 25]. In that context, abdominal complications [26], including hepatic, pancreatic and gastrointestinal malignancy [27–30], are increasing. Monitoring of abdominal complications of CF [31] could be improved with native MRI diagnostics, including DWI [32].

To date, DWI SMS Moco has been used primarily to evaluate adult patient with liver metastasis from neuroendocrine tumors (NET) [33], and DWI SMS Moco has achieved higher spatial resolution and has been shown to be accurate in identifying lesions [33].

In this study, we compare the scan time, the image quality, SNR and ADC values of DWI SMS Moco to sDWI in free-breathing abdominal MRI in pediatrics and young adolescent patient with CF.

## Materials and methods

### Study design

To report and publish the study results of this prospective single-center observational study all patients, adults and, in case of children and adolescents, their custodial parents needed to give written informed consent after they were informed about the MRI examination. Approval from local Ethics Committee was obtained and the study was registered in the national trials register. We examined consecutively CF patients between November 2020 and March 2021. MRI of the upper abdomen was indicated clinically within the scope of evaluation for abdominal pain.

### Image acquisition

MRI was performed on a 1.5 T system (MAGNETOM Aera; Siemens Healthcare, Erlangen, Germany) using a vendor-supplied 18-channel anterior surface body coil, in combination with 32-channel spine coil. All patients received a standard native upper abdominal examination protocol with coronal and transversal half-Fourier acquisition single-shot turbo spin echo imaging (HASTE), transversal T_1_-weighted imaging with water-fat separation based on the Dixon method, and diffusion-weighted sequences (DWI). Transversal DWI of the upper abdomen included two different acquisition protocols in free-breathing technique without navigator-controlled triggering. Both, the sDWI and DWI SMS Moco protocols were identical, except for the latter using SMS and motion correction and for some parameters like the number of slices concatenations being allowed to vary in order to compensate for the reduced repetition time (TR) enabled by SMS. As all examinations were clinically indicated, some variations in the protocols were allowed. In particular, the protocols were adapted to the different patient sizes. The integrated image processing in the DWI SMS Moco prototype focuses on a better alignment and combination of the multiple repetitions acquired in conventional DWI that originate from the different diffusion directions, diffusion weightings as well as the averages to increase SNR. Motion correction in DWI SMS Moco was implemented in the reconstruction environment of the scanner and executed as part of the image reconstruction. Once all averages for a given *b*-value and slice position were available, a keyframe was selected within this set by choosing the acquisition with the lowest overall standard deviation compared to all other images with the same *b*-value and slice position. After pairwise registering all images to this keyframe, averages with same diffusion direction were adaptively combined as complex-valued images and following magnitude extraction, trace-weighted images were calculated and then also pairwise registered using the lowest *b*-value as reference. This ensures that the trace-weighted images are also aligned before subsequently calculating the ADC maps. Detailed sequence parameters of the sDWI and the DWI SMS Moco are summarized in Table 1. Both measurements were performed using diffusion gradients applied in four directions with *b*-values of 50, 400 and 800 s/mm^2^.

**Table 1:** Diffusion-weighted imaging (DWI) sequence parameters

Sequence parameters	sDWI	DWI SMS Moco
Breathing scheme	Free-breathing	Free-breathing
Slice thickness/gap, mm	5/0.5	5/0.5
No. slices*	40	40
Bandwidth, Hz/pixel	1925	1925
TE, ms	66	67
TR*, ms and (concatenations)*	2600 (3), 7200 (1)	2000 (2)
FOV read, mm^2^	350, 400	350, 400
FOV phase, %	80	80
Matrix, read x phase	130 × 108	130 × 106
Voxel size, mm^3^	1.33 × 1.33	1.35 × 1.35
Scan time, s	308	199
Diffusion preparation * b*-values, s/mm^2^	50, 400, 800	
Averages per * b*-value	1, 2, 4	
Directions	4	
Flip angle, °	90	90
GRAPPA acceleration factor	2	2
SMS acceleration factor	–	2
Motion correction	–	On

Summary of sequence parameters for the standard DWI (sDWI) and DWI with simultaneous multi-slice and motion correction (DWI SMS Moco)

*TE* time of echo, *TR* time of repetition, *FOV* field of view, *GRAPPA* generalized autocalibrating partial parallel acquisition, *SMS* simultaneous multi-slice

*May vary depending on the number of acquired slices adapted to different patient size

### Assessment of image quality

For qualitative analysis, two pediatric radiologist, one 10 years of experience and the other 15 years in abdominal imaging (PCK and KG), evaluated the image quality of b800 image and ADC image of the sDWI and DWI SMS Moco independently. The readers were blind to patient and sequence data. Image quality of b800 images and corresponding ADC maps were rated on a 5-point Likert scale (1 not interpretable, 2 bad, 3 fair, 4 good and 5 very good). The following image quality criteria were assessed: overall image quality and delimitability of mesenteric lymph nodes.

### Quantitative assessment of SNR and ADC

The SNR and ADC values were determined by region-of-interest (ROI) assessment. For this purpose, a circular ROI with a maximum area of approximately 1 cm^2^ was manually positioned in 8 different representative localizations using Mint lesion™ software (Mint Medical GmbH, Heidelberg, Germany), a workflow optimized software solution. If the contiguous tissue of the corresponding structure was not large enough, the size of the ROI was adjusted accordingly. Furthermore, ROIs were located in homogeneous tissue regions so that the standard deviation of the individual ROI’s could be used as a noise measure for SNR calculation [34]. Measurements were made in parenchyma of left and right kidney, in liver segment V and IVa, in lower back muscles, in spleen and in head and tail sections of pancreas. Because of automatic conformity checks by the MINT lesion™ software, identical ROI positioning was ensured in the b800 image and ADC image in both the standard DWI and the DWI with SMS. For each ROI, mean value, standard deviation (SD) minimum and maximum value were recorded automatically by MINT lesion™ software.

### Statistical analysis

All statistical analyses were performed using the Python programming language (Python Software Foundation, https://www.python.org/) and the Statsmodels [35] and Pingouin packages [36].

Mean Ratings, ADC and SNR were expressed as mean and standard deviation. We used descriptive statistics to summarize the population characteristics and image findings. Image quality scores of standard and simultaneous multi-slice DWI were compared using a Wilcoxon signed-rank test. Interrater agreement was calculated by intraclass correlation (ICC).

## Results

### Clinical characteristics

A total of 16 children and young adolescents suffering CF were included in the study. The average acquisition time for free-breathing sDWI was 3:39 min ± 25 s, compared with 2:27 min ± 13 s for DWI SMS Moco. Further clinical parameters of all examined patients are summarized in Table 2.

**Table 2:** Patient characteristics of the study cohort

Marker	Data*
Female	9/16 (56.3)
CFTR gene type	
F508del/F508del	15/16 (93.8)
Other/other	1/16 (6.7)
Age, years	22 [20.4–26.2]
< 18	5/16 (31.2)
≥ 18	11/16 (68.8)
Weight, kg	57.4 [49.4–58.4]
Height, m	1.6 [1.6–1.7]
Body mass index, kg/m^2^	19.5 [18.7–20.8]
Pancreatic insufficiency, yes	16/16 (100)
CFRD, yes	8/16 (50)
CFLD, yes	8/16 (50)
FEV1, %	72.9 [65.7–73.5]
FVC, l	3.4 [3.2–3.9]

*CFTR* cystic fibrosis transmembrane conductance regulator; *CFRD* cystic fibrosis related diabetes; *CFLD* cystic fibrosis liver disease; *FEV1* forced expiratory pressure in 1 s; *FVC* forced vital capacity

*Categorical data are presented as n/N (%), and numerical data are presented as median [IQR, 25th–75th percentile]

### Qualitative analyses—subjective image quality

#### Intraclass correlation ICC

Assessing the reliability of ratings by a two-way mixed intraclass correlation model pointed to a high correlation between the two raters' quantitative assessments. The high ICC of 0.71 to 1.0 in the observations indicates the low variance between the assessment values of the two readers. Table 3 shows the ICC values of the observed categories image quality and delimitability of the mesenteric lymph nodes, respectively in the b800 image and in the ADC map of sDWI and DWI SMS Moco.

**Table 3:** Intraclass correlation (ICC) to quantify interrater reliability between two raters

Categories	ICC	95% CI
Image quality b800		
sDWI	0.91	0.74, 0.97
DWI SMS Moco	0.83	0.5, 0.94
Image quality ADC		
sDWI	0.9	0.71, 0.96
DWI SMS Moco	1.0	0.88, 0.99
Delimitability of the mesenteric lymph nodes b800		
sDWI	0.93	0.79, 0.97
DWI SMS Moco	0.86	0.6, 0.95
Delimitability of the mesenteric lymph nodes ADC		
sDWI	0.87	0.61, 0.95
DWI SMS Moco	0.71	0.17, 0.9

*ICC* intraclass correlation: ICC3k average fixed raters

### Mean ratings

**Fig. 1:**
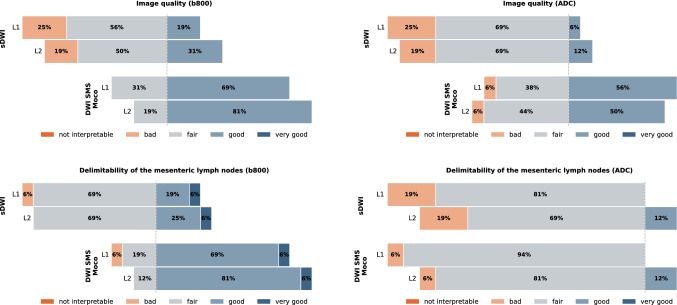
Qualitative analysis of the mean ratings of reader 1 (L1) and 2 (L2) for standard diffusion weighted imaging (sDWI) and diffusion-weighted imaging with simultaneous multi-slice and motion-correction (DWI SMS Moco) for the *b*-values of 800 (b800) and the corresponding apparent diffusion coefficient (ADC) map in both categories: image quality and delimitability of the mesenteric lymph nodes

The mean ratings of DWI SMS Moco were classified better in all four categories compared to the sDWI. These four categories were overall image quality in *b*-values of 800 (b800) (3.75 ± 0.43 vs 3.03 ± 0.68, *p* < 0.01), overall image quality in ADC map (3.47 ± 0.61 vs 2.88 ± 0.54, *p* < 0.05), delimitability of the mesenteric lymph nodes in b800 (3.84 ± 0.57 vs 3.31 ± 0.63, *p* < 0.05), and delimitability of the mesenteric lymph nodes in ADC map (3 ± 0.35 vs 2.88 ± 0.48, *p* < 0.05) (Table 4 and Fig. 1). Due to the high agreement of the values of the intraclass correlation of the interrater variability between reader 1 and 2, the values for the mean rating were combined.

**Table 4:** Mean ratings of qualitative analyses for sDWI and DWI SMS Moco

Imaging parameters	sDWI	DWI SMS Moco	*p*-value overall
Image quality b800	3.03 ± 0.68	3.75 ± 0.43	< 0.01*
Image quality ADC	2.88 ± 0.54	3.47 ± 0.61	< 0.05*
Delimitability of the mesenteric lymph nodes b800	3.31 ± 0.63	3.84 ± 0.57	< 0.05*
Delimitability of the mesenteric lymph nodes ADC	2.88 ± 0.48	3 ± 0.35	< 0.05*

Mean value data are presented with simple standard deviation (SD), *p*-values for the overall comparisons using the Wilcoxon signed-rank test, *p* < 0.05 is significant, *statistically significant

**Fig. 2:**
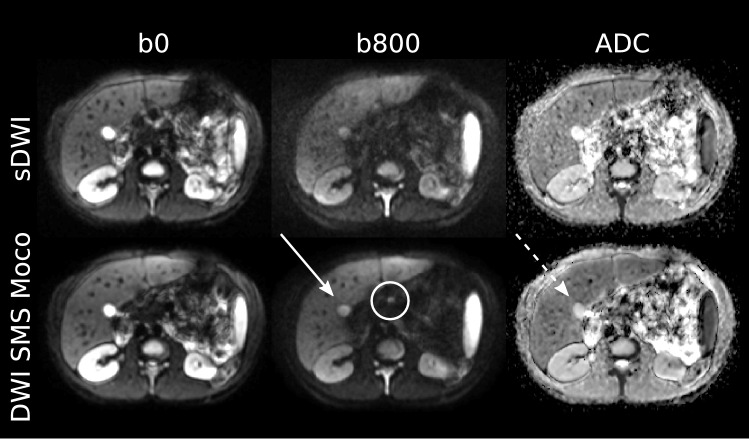
Comparison of image quality of sDWI and DWI SMS Moco in an 18-year-old patient with cystic fibrosis (CF), pancreas fibrosis and microgallbladder. Images show the acquired DWI images with *b*-values of 0 (b0) and 800 (b800) as well as the corresponding ADC map for both acquisitions. Shown is the sharp boundary of the microgallbladder in the b800 image (white arrow) of the DWI SMS Moco and the corresponding T2 shine-through effect in the ADC map (white dashed arrow). Further, a better differentiability of very small mesenteric lymph nodes in the DWI SMS Moco ventral to the thinned fatty pancreas in the b800 image can be observed (white circle)

**Fig. 3:**
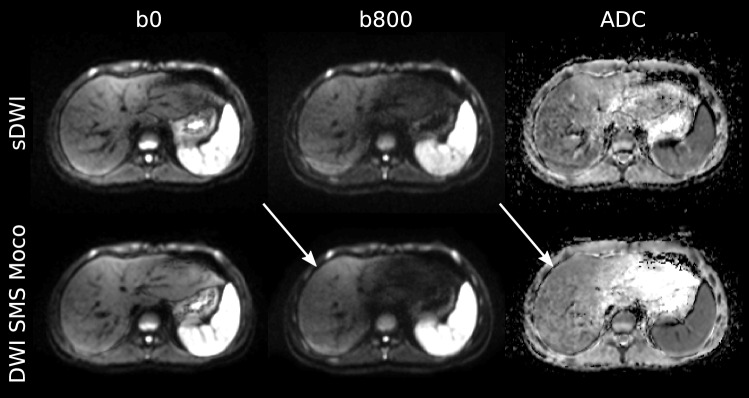
Example of an adolescent patient. 14-year-old male patient with CF, cystic fibrosis related diabetes (CFRD) and cystic fibrosis related liver disease (CFRLD). Images show the acquired DWI images with *b*-values of 0 (b0) and 800 (b800) as well as the corresponding ADC map for both acquisitions of sDWI and DWI SMS Moco. Improved image quality with homogeneous image impression of the liver parenchyma in the b800 image and ADC map in DWI SMS Moco compared to standard DWI (white arrows)

Both readers classified the image quality in the b800 image and the ADC map both in the sDWI and in the DWI SMS Moco as predominantly good or fair and rather rarely in the sDWI (b800 and ADC map) and DWI SMS Moco (ADC map) as bad, and none of the cases as not interpretable. The DWI SMS Moco showed only fair or good in the b800 image regarding image quality (Figs. 1, 2 and 3).

### Quantitative analysis

#### SNR

**Fig. 4:**
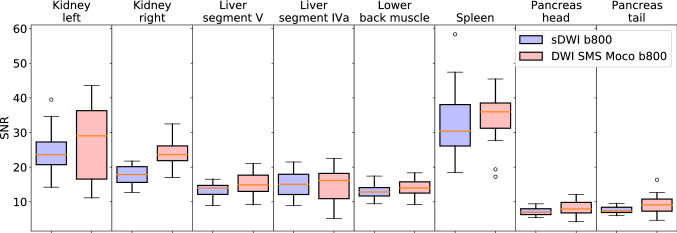
Boxplots of SNR measured in 8 different locations (renal parenchyma of the left and right kidney, liver parenchyma in segment V and IVa, lower back muscle, parenchyma of spleen, pancreas in head and tail) for sDWI (blue) and DWI SMS Moco (rose)

**Fig. 5:**
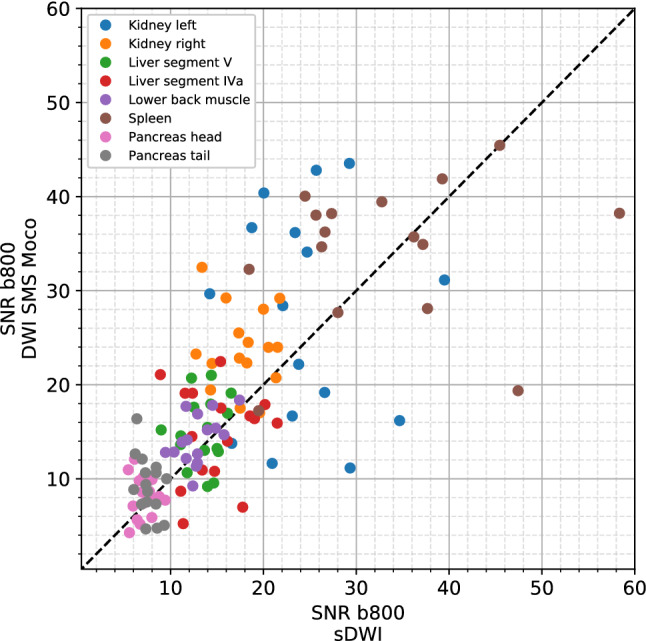
Scatterplot of all SNR values measured in *b*-values of 800 (b800) comparing sDWI with DWI SMS Moco

The SNR showed no significant difference in 7 out of 8 locations in the comparison between sDWI and DWI SMS Moco. In the parenchyma of the right kidney, the SNR of the DWI SMS Moco was significantly better than in the standard DWI (23.9 ± 4.1 versus 17.8 ± 2.9, *p* < 0.01) (Figs. 4 and 5, Table 5).

**Table 5:** SNR values for sDWI and DWI SMS Moco

Localizations	sDWI	DWI SMS Moco	*p*-value (sDWI vs. DWI SMS Moco)
Kidney left	24.5 ± 6.2	27.1 ± 11.0	0.55
Kidney right	17.8 ± 2.9	23.9 ± 4.1	≤ 0.01*
Liver segment V	13.6 ± 2.0	15.0 ± 3.5	0.2
Liver segment IV a	15.0 ± 3.6	14.8 ± 4.9	0.94
Lower back muscle	12.9 ± 2.0	14.2 ± 2.5	0.19
Spleen	33.2 ± 10.5	34.2 ± 7.5	0.43
Pancreas head	7.1 ± 1.1	8.1 ± 2.1	0.17
Pancreas tail	7.6 ± 1.0	9.2 ± 3.1	0.07

SNR values and SD for 8 different localizations in the abdomen (*n* = 16), *p* < 0.05 is significant, *statistically significant

#### ADC values

**Fig. 6:**
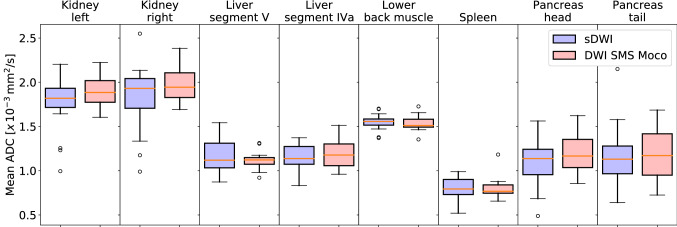
Boxplots of ADC values measured in 8 different localizations (renal parenchyma of the left and right kidney, liver parenchyma in segment V and IVa, lower back muscle, parenchyma of spleen, pancreas in head and tail) in sDWI (blue) and DWI SMS Moco (rose)

**Fig. 7:**
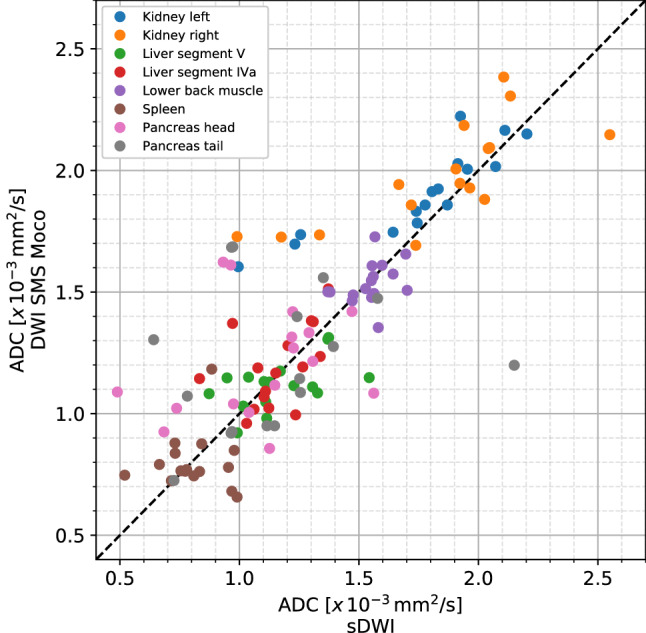
Scatter plot of all ADC values measured in 16 patients comparing sDWI with DWI SMS Moco

The ADC values (in × 10^−3^mm^2^/s) showed no significant difference between sDWI and DWI SMS Moco in the eight different locations (Figs. 6, 7 and Table 6).

**Table 6:** ADC values for sDWI and DWI SMS Moco

Localizations	sDWI	DWI SMS Moco	*p*-value (sDWI vs. DWI SMS Moco)
Kidney left	1.75 ± 0.32	1.91 ± 0.17	0.28
Kidney right	1.83 ± 0.38	1.98 ± 0.20	0.35
Liver segment V	1.16 ± 0.18	1.12 ± 0.10	0.62
Liver segment IVa	1.16 ± 0.14	1.19 ± 0.16	0.75
Lower back muscle	1.55 ± 0.09	1.54 ± 0.08	0.55
Spleen	0.81 ± 0.12	0.8 ± 0.12	0.61
Pancreas head	1.09 ± 0.28	1.21 ± 0.22	0.32
Pancreas tail	1.16 ± 0.36	1.21 ± 0.28	0.65

ADC values in × 10^−3^mm^2^/s and SD for 8 different localizations in abdomen (n16)

## Discussion

DWI SMS Moco is highly suited for pediatric and young adult patients with cystic fibrosis. It is faster than and superior in image quality compared to standard DWI in abdominal imaging.

Various studies reviewed the application of DWI with SMS technique in whole-body [37], prostate [38], rectum [39] and breast [40–42]. Almost exclusively, these studies refer to adult patients and healthy volunteers. For the first time, we investigated in our study DWI with SMS technique in pediatric and young adult patients with cystic fibrosis.

The simultaneous excitation of multiple slices shortens the scan-time of the diffusion-weighted sequence and improves the performance of the MRI examination of the abdomen [43, 44]. The faster acquisition time makes the examination more comfortable and reduces artefacts due to motion in the bowel or general restlessness. This is an advantage for patients who cannot lie still for long such as, especially, pediatric patients, severely ill patients and patients with disturbed breathing like patients suffering from cystic fibrosis.

Our results showed that the acquisition time of DWI using SMS and Moco is 32% faster than standard DWI. The reduction of the acquisition time of DWI in SMS technique has already been shown by several studies including whole-body imaging and different regions and organs of the abdomen as liver, spleen, pancreas and pelvic lymphnodes [15, 43, 45–48]. Thus, our results are in accordance with a recently published study by Tabari et al. [49]. This study examined 33 children and adolescents with tuberous sclerosis complex (TSC) with DWI and SMS in abdominal MRI to detect and monitor focal solid and cystic renal lesions. This study revealed a 55% reduction in scan time. But the image quality was only approximately identical [49]. Recently, Boss et al. investigated in their study ten healthy volunteers underwent DWI of the upper abdomen at 3 T. They were able to reduce scan time approximately 45% with DWI and SMS without reducing SNR but with minimally reduced image quality at the liver dome [16].

In contrast, in our study the image quality by using motion correction in the DWI with SMS was improved significantly. Both, the overall image quality and the delimitability of mesenteric lymph nodes were better using DWI with SMS and motion correction compared to the standard DWI. So, DWI with SMS and motion correction seems to be a powerful modern diffusion-weighted sequence for abdominal imaging which may be helpful especially in children with reduced compliance. There is only one comparable paper in the literature so far by Xu et al. [33]. These authors used MRI to examine the liver of 15 adult patients with metastases of a neuroendocrine tumor. They were able to demonstrate that DWI with SMS and motion correction had significantly higher overall image quality and significantly fewer artefacts than conventional DWI. The observed excellent delimitability of lymphnodes in our study could help to better visualize inflammatory activity in the abdomen, especially for CF patients [50]. According to a study by Meeker et al. using a colonized CF mouse model, mesenteric lymph nodes (MLN) seem to play an important role regarding to the adaptive immune response in inflammatory processes in the gut [51]. Additionally, Radmard et al. could demonstrate in patients with Crohn’s disease (CD) that the ADC value of MLN could predict disease activity [52]. Therefore, additional abdominal MRI examinations with DWI and SMS and Moco in CF patients seem promising, especially for monitoring the response to therapy with modern highly effective modulators [53].

In the quantitative analysis, the signal-to-noise ratio of the DWI with SMS and motion correction compared to the standard DWI was identical in 7 of 8 localizations and even significantly higher in the parenchyma of the right kidney. Moreover, our results showed no significant differences in ADC values between DWI with SMS and motion correction compared to standard DWI. This is in contrast to some previous studies [33, 43]. Several authors have demonstrated significantly lower ADC values in DWI with SMS compared to standard DWI [43, 54, 55]. In contrast, Xu was able to show significantly higher ADC values in the DWI with SMS and motion correction compared to the standard DWI [33]. Therefore, our result is not consistent with the results of Taron and Xu [33, 43]. A possible explanation for this could be the minimal difference in repetition time (TR) between standard DWI and DWI with SMS and motion correction [55] for most of the used protocols. In our study, the median TR for the standard DWI was 2600 and for the DWI with SMS and motion correction 2000. As Taron et al. already explained in their study, a low TR can lead to a signal reduction due to T_1_ saturation effects in the higher *b*-values [56]. This is important to consider with DWI SMS as application of SMS only leads to a reduction of scan time if the TR is reduced accordingly. As shown by Ogura et al. in phantom measurements at 3 Tesla tissues with a long T_1_ could produce erroneous ADC values when acquired with very short TR [57]. As also demonstrated by Ogura et al. such effects become most significant when the TR is becoming shorter or close to the T_1_ relaxation time of the tissue of interest. Reference T_1_ values for the tissues of interest in this study are in the range between 700 and 1400 ms and thus smaller than the TR of the SMS sequence [58]. Most importantly, we have shown that when keeping TR comparable between non-SMS and SMS acquisition the same ADC values are obtained. Obtaining reliable ADC values is an important tool for tissue characterization in modern MRI imaging for monitoring and differentiating between various tumors and inflammations [59–62]. For 2D multi-slice acquisitions, we thus recommend adjusting the number of slice concatenation, i.e. the segmentation of the slice stack into multiple blocks, to avoid a significant reduction of the TR when using SMS.

**Fig. 8:**
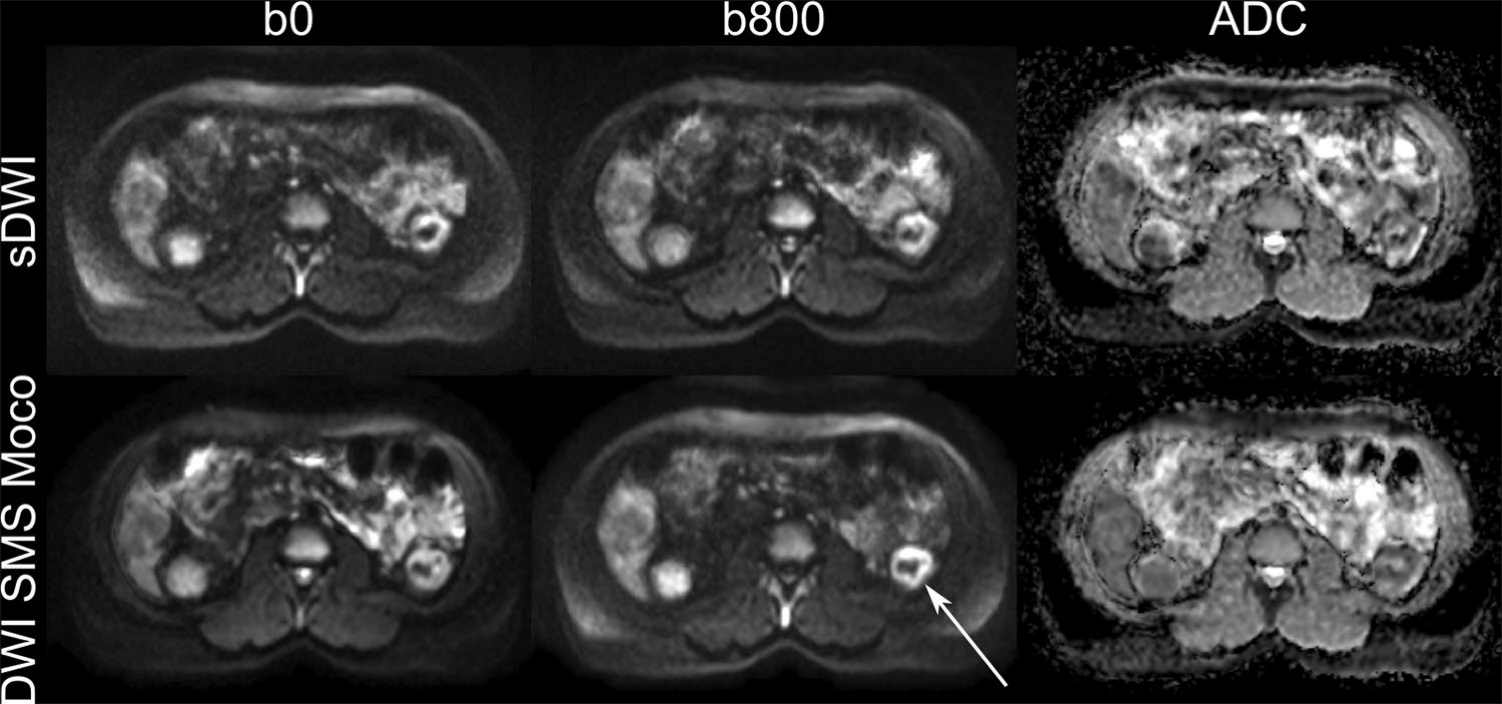
Example of an adult patient. 42-year-old female patient with CF, metastatic colon carcinoma, cystic fibrosis related diabetes (CFRD) and cystic fibrosis related liver disease (CFRLD). Clearly recognizable circular diffusion restriction in the descending colon (white arrow) in the b800 image in DWI SMS Moco, corresponding to histologically confirmed adenocarcinoma

Consequently, SMS DWI Moco could be an interesting approach for further studies on MRI examinations of the abdomen in young CF patients under HEM [63]. The pancreas and liver of young CF patients can show early changes with fatty deposits, fibrosis and remodeling processes due to the chronic inflammation. This increases the risk of malignant neoplasms in the liver and pancreas [64, 65]. Using DWI with SMS and motion correction as a high-resolution MRI sequence with shorter acquisition time in free breathing could significantly increase the sensitivity for detecting suspicious lesions (Fig. 8).

There are several limitations in this study. First, a certain disadvantage of the study is the small number of 16 patients examined. Second, similar to Xu's work, we acquired both standard and DWI with SMS and motion correction in free breathing. There was no comparison of diffusion-weighted sequences with other breathing schemes as respiratory-triggered mode or breath-hold mode. Because CF patients with pulmonary complications sometimes have very shallow and irregular breathing, an additional sequence with respiratory navigation, which has a significantly longer scan time, or another sequence in breath-hold technique would not have been feasible for the patients. Therefore, we deliberately avoided such additional sequences in this study. Third, this study focused only on DWI images and thus did not allow for comparison regarding diagnostic performance between DWI and other non–DWI sequences. Further studies should explore such comparisons.

## Conclusion

DWI with SMS and motion-correction can provide better overall image quality and delimitability of mesenteric lymph nodes in shorter acquisition time to standard DWI in an examination protocol for abdominal MRI. Therefore, DWI SMS Moco could improve preventive abdominal MRI as a quick, safe and non-invasive approach to evaluate effects in pediatric and young adolescent CF patients under highly effective modulator therapy.

## Data Availability

The datasets generated during and/or analyzed during the current study are not publicly available, but are available from the corresponding author on reasonable request.

## Abbreviations

IQ: Image quality
SNR: Signal-to-noise
ADC: Apparent diffusion coefficient
DWI SMS Moco: Diffusion-weighted imaging with simultaneous multi-slice and motion-correction
sDWI: Standard diffusion weighted imaging
CF: Cystic fibrosis
MRI: Magnetic resonance imaging
CNS: Central nervous system
WBI: Whole-body imaging
CFTR: Cystic fibrosis transmembrane conductance regulator
HEM: Highly effective modulator
SMS: Simultaneous multi-slice
ROI: Region-of-interest
CFRD: Cystic fibrosis related diabetes
CFRLD: Cystic fibrosis related liver disease
